# Statistical issues in trials of preexposure prophylaxis

**DOI:** 10.1097/COH.0000000000000218

**Published:** 2015-10-28

**Authors:** David T. Dunn, David V. Glidden

**Affiliations:** a MRC Clinical Trials Unit at UCL, London, UK; b University of California, San Francisco, California, USA

**Keywords:** intention-to-treat, noninferiority, open-label, placebo-controlled, risk compensation

## Abstract

**Purpose of review:**

We discuss selected statistical issues in the design and analysis of preexposure prophylaxis (PrEP) trials. The general principles may inform thinking for other interventions in HIV prevention.

**Recent findings:**

To date, four different designs have been used to determine the effectiveness of PrEP: randomized, double-blind, placebo-controlled; randomized, open-label, immediate or delayed access; nonrandomized comparison of HIV incidence according to the level of drug detected; comparison of the observed HIV incidence to the expected rate using historical control data. Open-label trials of PrEP, which assess public health effectiveness, complement the placebo-controlled trials which established the biological efficacy of TDF/ FTC. Future trials of PrEP will be highly challenging to design since a no PrEP group is difficult to justify and the natural control regimen, TDF/FTC, is highly efficacious.

**Summary:**

Standard statistical paradigms for noninferiority trials should be reconsidered for evaluating alternative PrEP regimens.

## INTRODUCTION

Randomized controlled trials of preexposure prophylaxis (PrEP) have given rise to specific statistical challenges both in design and analysis. In this article we focus in depth on three issues: assessing the influence of risk compensation, dealing with patients with acute HIV infection at study enrolment, and the design of future studies in the context of a highly efficacious preexisting regimen.  

### Risk compensation and the limitation of placebo-controlled trials

‘Risk compensation’ is the adjustment of behaviour in response to a perceived reduction in risk, a critical issue in the public health implementation of PrEP because of the potential for increased risky sexual behaviour which could counteract biological efficacy [1]. Placebo-controlled randomized trials are regarded as the gold standard for establishing the biological efficacy of an experimental drug. A key rationale for using placebo in trials of PrEP agents has been to avoid bias because of differential exposure to HIV caused by different sexual behaviour in the randomized groups; this contrasts with the real-life situation, where individuals know if they are taking an active drug. A frequently unappreciated point is that risk compensation cannot be assessed by standard within or between group comparisons in a placebo-controlled trial [2]. The European Medicines Agency stated that ‘The behavioural impact of PrEP on risk compensation and condom replacement cannot be assessed in prelicensure placebo-controlled trials’ and that ‘it is mandatory that the marketing authorisation application contains a risk management plan that adequately covers the public health impact of the PrEP intervention’ [3].

In an imaginative analysis to gain insights into risk compensation in the Preexposure Prophylaxis Initiative (iPrEx) trial, Marcus *et al.*[4] compared patients who believed they were taking active drug (*n* = 553) with patients who believed they were taking placebo (*n* = 223). Patients who believed they were receiving active drug had higher number of receptive partners at baseline, but the difference between the two groups did not increase during follow-up after study drug was initiated. There was also no difference at any time point in the percentage of receptive anal intercourse partners using condoms. These results were interpreted as no evidence of risk compensation. However, this study has several limitations: confidence intervals were relatively wide (the analysis excludes 1429 patients who did not predict their treatment assignment); the accuracy of self-reported data on sexual practices; and the fact that groups were based on perceived assignment rather than certain knowledge as pertains in real-life. A further limitation is that risk compensation is a function of how effective an individual considers the intervention to be, and the very high biological efficacy of tenofovir disoproxil fumarate/emtricitabine (TDF/FTC) was not known at the time the study was conducted.

Grant *et al.*[5^▪^] assessed and presented a detailed analysis of a cohort study of MSM enrolled from three previous randomized controlled trials of PrEP (including iPrEx) that were offered open-label PrEP. The authors assessed risk compensation by looking at longitudinal changes in behaviour, comparing patterns among men who accepted the offer of PrEP and those who declined it. Self-reported total number of sexual partners, noncondom receptive/ insertive anal intercourse decreased during follow-up in both groups and to a similar extent. Syphilis incidence was also similar in the two groups. However, the fact that the control group was not randomized limits the interpretability of these data.

The most robust data on risk compensation to date were obtained in PROUD, a pragmatic, open-label trial which attempted to mimic how PrEP would be administered in routine clinical practice [6^▪▪^]. Eligible patients were randomized to receive daily TDF/FTC either immediately (*n* = 275) or after a deferred period of 1 year (*n* = 269). Data from the first year of follow-up allowed direct assessment of risk compensation. Patients were asked to complete monthly questionnaires and daily diaries about sexual behaviour but the completion rates of these were low, particularly in the deferred group. Accordingly the investigators reported cross-sectional analyses of sexual behaviour based on baseline and 1 year questionnaires only. No differences were found in terms of the total number of different anal sex partners but there was marginal evidence of a larger proportion of PrEP recipients at 1 year who reported receptive anal sex with 10 or more partners without a condom. An indirect, but more objective measure of risky sexual behaviour, is the diagnosis of other sexually transmitted infections (STIs) [7]. PROUD reported a slightly higher rate of diagnosis with any bacterial STI in the immediate PrEP group (57%) than in the deferred group (50%). However, after adjustment for the number of screens, there was no evidence of a difference between the groups in the frequency of bacterial STIs, either individually or overall.

A potentially important effect which could impact negatively on the cost–effectiveness of PrEP is that some men who have been using condoms consistently may stop doing so because they are able to access PrEP. Such men have not been eligible for PrEP trials to date and are unlikely to be formally eligible in PrEP implementation programmes. However, setting rigid criteria for PrEP access is not realistic, and if this phenomenon is real it will be difficult to detect it.

**Box 1:**
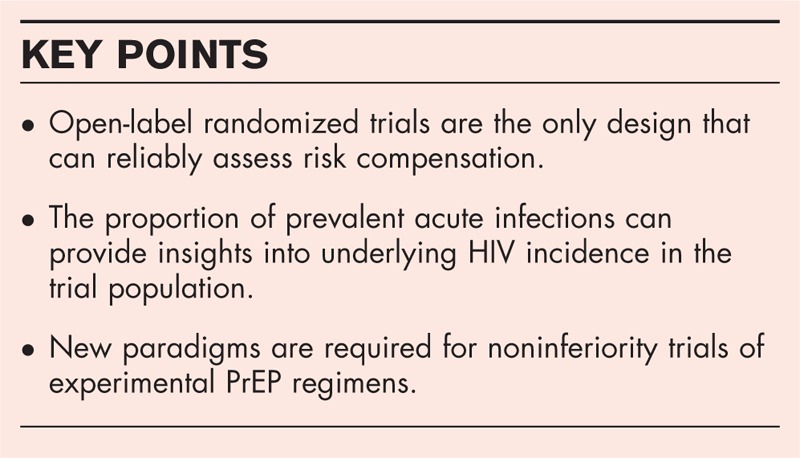
no caption available

### Acute HIV infections at study enrolment in the analysis

A clinical challenge with PrEP is the window period between exposure to HIV and the (assay-dependent) detection of infection, meaning that PrEP is inevitably initiated in some individuals who are already infected. The procedure used in most trials has been to perform a point-of-care serological test for HIV on the day of enrolment and to store an additional plasma sample that is retrospectively tested for HIV RNA, the earliest marker for HIV infection, if the patient had a reactive HIV antibody test at their first (or early) follow-up visit [8–12]. In real-life clinical practice, procedures are usually less stringent than in trials. United States guidelines recommend ‘At a minimum, clinicians should document a negative antibody test result within the week before initiating (or reinitiating) PrEP medications’ [13]. Also, samples may not be routinely stored, precluding the possibility of retrospective testing.

The primary efficacy analyses of trials have generally excluded patients with detectable HIV RNA at enrolment [modified intention-to-treat, (mITT)] on the grounds that PrEP cannot possibly avert infection in these individuals. (PrEP may have a postexposure prophylaxis effect but only if initiated within 48–72 h of exposure.) From an effectiveness rather than an efficacy perspective a full ITT analysis including all patients is arguably the more relevant [14]. In particular, analyses of safety outcomes should be intention-to-treat (ITT), particularly those relating to drug resistance, as viral mutations are particularly likely to emerge during acute infection under selective drug pressure.

In practice, ITT and mITT analyses in most studies produce very similar results as the number of prevalent acute infections is generally much smaller than the number of incident infections. However, it can make a material difference in studies where adherence to PrEP is high. For example, of the five infections in the immediate PrEP arm in PROUD, two occurred at enrolment. Here, the estimated efficacy is 78% under an ITT analysis compared with 86% under an mITT analysis (Table 1). Note that there is little effect on the rate difference (or number-needed-to-treat), the most relevant measure for public health.

**Table 1:** Impact on effect measures of including or excluding patients with acute HIV infection at enrolment in the PROUD trial

	ITT (all patients)		Modified ITT (excluding acute cases)	
	Immediate	Deferred	Immediate	Deferred
No. of infections	5	21	3	20
Follow-up (person-years)	243.5	222.1	243.5	222.1
Incidence rate^*^	2.1	9.5	1.2	9.0
Rate difference^*^	7.4		7.8	
Number needed to treat	13.5		12.9	
Efficacy (%)	78		86	

Three patients (two Immediate, one Deferred) tested nonreactive by a third-generation rapid test on the day of enrolment but reactive with a joint antigen/antibody assay. ITT, intention to treat.

**FIGURE 1:**
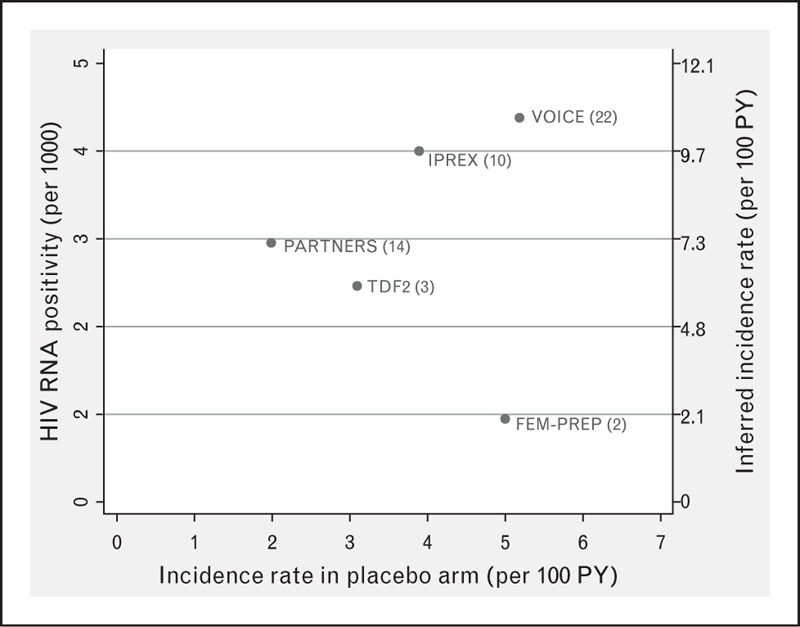
Proportion of patients with acute infection at enrolment plotted against incidence of infection observed in placebo recipients. Limited to studies that tested enrolment plasma samples retrospectively for HIV RNA. Value in brackets is number of acute infections observed in each study. The inferred incidence of infection at enrolment (plotted on right-hand vertical axis) was estimated by dividing the proportion of patients who were HIV RNA-positive/antibody negative by the mean interval between the detection of HIV RNA and antibody [20]. We assumed a value of 15.1 days using data from Eller *et al*. [21].

Finally, in the following section we raise the possibility of using the number of prevalent acute infections (antibody negative/HIV RNA-positive result on enrolment sample) to measure the underlying ‘force of infection’ in the trial population. The method is described in the footnote to Fig. 1, which shows the inferred baseline incidence plotted against the observed incidence of infection among patients who were allocated to placebo in trials that tested enrolment samples for HIV RNA. With one exception, it over-estimates incidence is overestimated by a factor of between 2 and 3. There are two main possible explanations for this. First, the calculation is highly sensitive to the assumption about the mean interval between detectable circulating viral RNA and detectable circulating antibody, which may be incorrect. Second, patients may have been motivated to join the trial because they had recently been at especially high risk of exposure to HIV. Nonetheless, in large trials this approach can provide a rough estimate of the underlying rate of infection.

### Future studies and the challenge of a highly-efficacious control regimen

Although TDF/FTC is the only drug currently approved by Food and Drug Administration for prevention, there is a pipeline of other agents, particularly long-acting agents [15]. Given the proven biological efficacy of TDF/FTC, there are ethical barriers to conducting future clinical trials that include a no PrEP comparison group. Possible exceptions to this are populations where PrEP is not policy or where adherence to daily TDF/FTC is uncertain.

Donnell *et al.* comprehensively reviewed study designs for PrEP interventions, assuming daily TDF/FTC to be the control regimen [16^▪▪^]. They considered three different experimental regimens: a new daily drug, a long-acting drug, and a different TDF/FTC dosing strategy. For the first of these scenarios, a noninferiority design would be the natural choice. The study explored noninferiority margins of 1.10, 1.20, and 1.25 on a hazard ratio scale. For the highest noninferiority margin of 1.25, and assuming the experimental intervention to be equally effective to TDF/FTC, the authors show that a trial would have to accumulate a total of 844 HIV events to be sufficiently powered; this translates to a sample size of approximately 19 000 subjects for HIV incidence of 2.25/100 person-years and 2 years follow-up on average – an infeasible undertaking.

Further calculations were made under the assumption that the experimental agent is more effective than TDF/FTC, to enable smaller, more realistic sample sizes. However, in the face of strong evidence that TDF/FTC confers very high protection if adequate drug concentrations are achieved [17], this assumption is plausible only in comparisons with long-acting drugs in a population likely to experience barriers to adherence to a daily oral medication.

The large number of required events for noninferiority studies is driven mainly by the use of the hazard ratio (which is based on the multiplicative scale) for assessing noninferiority. From a public health perspective, the rate difference is the more important metric as it translates directly to the number needed to treat [18], and this concept can be utilized in the comparison of drugs as well as to a comparison of drug versus no treatment.

Suppose we did a clinical trial to compare an experimental preventive intervention (E) to daily TDF/FTC (control, C) in a group of 5000 volunteers. The trial randomizes 2500/arm and follows them for a total of 2 years, yielding the results in Table 2. The HIV rate ratio (relative to C) is 1.88 [95% confidence interval (CI) 0.74, 5.1]. Thus, the rate of HIV could be as much as five times higher for E and would clearly exceed any noninferiority margin. The rate difference is much narrower: 1.4 (95% CI −0.4 to 3.3)/1000 person-years. For every thousand people getting E rather than C for 1 year, the best estimate is that there would be 1.4 more infections (or 3.3 at most).

**Table 2:** Outcomes in hypothetical study – low-incidence population

Group	Total follow-up	HIV infections	Rate (per 1000 PY)	Effectiveness compared to N (%) (95% CI)
Control (C)	5000	8	1.6	60 (5, 85)
Experimental (E)	5000	15	3.0	25 (−54, 64)
No treatment (N)	5000	20	4.0	–

CI, confidence interval.

We now argue that information on the number of infections under the condition of no-treatment (N) is essential context, noting this group is not actually observed. Suppose, HIV incidence under N is 4.0/1000 person-years. The effectiveness of E compared to N is 25% (95% CI −54% to 64%) and the effectiveness of C compared to N is 60% (5–85%). It is helpful to compare the effectiveness estimate for E and C on the additive scale: 60 − 25% = 35% (95% CI −14 to 84%), which represents that proportional increase in the number of infections using E rather than C relative to the number of infections in the absence of PrEP. Thus given 5000 person-years follow-up we would expect 20 infections with no PrEP and 7 (15–8) more infections with the use of E rather than C (7/20 = 35%); this would seem to represent an appreciable loss of efficacy.

Consider an alternative scenario where the trial population is at 10 times higher risk of HIV and is highly adherent to both E and C (Table 3). Under this scenario, the effectiveness of E compared to N is 93% (95% CI 87–96%) and the effectiveness of C compared to N is 96% (92–98%). The HIV rate ratio is unchanged (1.88 = (1–93%)/(1–96%)), but the difference in effectiveness on the additive scale is much smaller: 96 − 93% = 3% (95% CI −1 to +8%). Given 5000 person-years follow-up, we still expect seven more infections with the use of E rather than C but this time against a background of 200 infections in the absence of PrEP. In this scenario, E would seem to be an acceptable alternative to C.

**Table 3:** Outcomes in hypothetical study – high-incidence population

Group	Total follow-up	HIV infections	Rate (per 1000 PY)	Effectiveness compared to N (%) (95% CI)
Control (C)	5000	8	1.6	96 (92, 98)
Experimental (E)	5000	15	3.0	93 (87, 96)
No treatment (N)	5000	200	40.0	–

PY, person-years.

The fact that underlying HIV incidence as well as adherence to PrEP can vary greatly between populations implies the need to anchor any comparison to the number of HIV infection we would have observed in the absence of PrEP. We propose, for wider discussion, the use of a two-part noninferiority definition: 


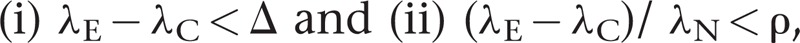


where *λ*_E_, *λ*_C_, and *λ*_N_ are estimates of HIV incidence in the E, C, and N groups respectively and the noninferiority margins (Δ, ρ) are appropriately chosen. (To simplify exposition, we have avoided attaching probabilistic statements to the lower confidence limits.)

For instance, in the low-incidence scenario the upper CI for *λ*_E_ − *λ*_C_ is 3.3/1000 and the upper bound on (*λ*_E_ − *λ*_C_)/*λ*_N_ is 0.84 (or 84% more of total infections). In the high-incidence scenario, the upper CI for *λ*_E_ − *λ*_C_ remains 3.3/1000 whereas the upper bound on (*λ*_E_ − *λ*_C_)/*λ*_N_ is now 0.08 (or 8% more of total infections). The first part of the definition is fully rigorous is the sense that it is intention-to-treat and does not rely on an external estimate of λ_N_, but this is required for the second part of the definition. The Partners Demonstration project estimated this based on the placebo rate of HIV in the cohort prior to the treatment period [19]. An alternative approach could be to use the proportion of patients with HIV RNA detected in their enrolment sample, as described earlier. A final possibility is to use external data in the population from which the study patients are recruited, although this can be misleading. The PROUD study observed an HIV incidence of 9.0/100 person-years in the deferred group, which was approximately seven-fold higher than a national estimate of 1.34/100 person-years for MSM attending sexual health clinics [6^▪▪^]; this underscores that it may be difficult to assemble control groups that accurately reflect the HIV risk of individuals who seek participation in a trial.

## CONCLUSION

Placebo-controlled and open-label trials of PrEP have addressed fundamentally different questions. The former evaluates the biological efficacy of the PrEP agent studied; the latter attempts to evaluate real-life effectiveness, reflecting the impact of risk compensation and actual adherence. Future trials of PrEP are highly challenging to design since daily TDF/FTC, the natural control regimen, is highly efficacious. New statistical paradigms for noninferiority trials are required, with statisticians and expert clinicians working closely together to develop these.

## Acknowledgements

None.

### Financial support and sponsorship

None.

### Conflicts of interest

There are no conflicts of interest.

## REFERENCES AND RECOMMENDED READING

Papers of particular interest, published within the annual period of review, have been highlighted as:

▪ of special interest
▪▪ of outstanding interest
